# Age-related alterations of articular cartilage in pituitary adenylate cyclase–activating polypeptide (PACAP) gene–deficient mice

**DOI:** 10.1007/s11357-019-00097-9

**Published:** 2019-10-26

**Authors:** Vince Szegeczki, Balázs Bauer, Adél Jüngling, Balázs Daniel Fülöp, Judit Vágó, Helga Perényi, Stefano Tarantini, Andrea Tamás, Róza Zákány, Dóra Reglődi, Tamás Juhász

**Affiliations:** 1 Department of Anatomy, Histology and Embryology, Faculty of Medicine, University of Debrecen, Nagyerdei krt. 98, Debrecen, 4032 Hungary; 2 Department of Anatomy, PTE-MTA PACAP Research Team, University of Pécs Medical School, Szigeti út 12, Pecs, 7624 Hungary; 3 Department of Geriatric Medicine, Reynolds Oklahoma Center on Aging, University of Oklahoma Health Sciences Center, Oklahoma City, OK USA

**Keywords:** Aggrecan, Collagen expression, Sox9, Sox5, Sox6, Hyaluronic acid

## Abstract

Pituitary adenylate cyclase activating polypeptide (PACAP) is an evolutionarly conserved neuropeptide which is produced by various neuronal and non-neuronal cells, including cartilage and bone cells. PACAP has trophic functions in tissue development, and it also plays a role in cellular and tissue aging. PACAP takes part in the regulation of chondrogenesis, which prevents insufficient cartilage formation caused by oxidative and mechanical stress. PACAP knockout (KO) mice have been shown to display early aging signs affecting several organs. In the present work, we investigated articular cartilage of knee joints in young and aged wild-type (WT) and PACAP KO mice. A significant increase in the thickness of articular cartilage was detected in aged PACAP gene–deficient mice. Amongst PACAP receptors, dominantly PAC1 receptor was expressed in WT knee joints and a remarkable decrease was found in aged PACAP KO mice. Expression of PKA-regulated transcription factors, Sox5, Sox9 and CREB, decreased both in young and aged gene deficient mice, while Sox6, collagen type II and aggrecan expressions were elevated in young but were reduced in aged PACAP KO animals. Increased expression of hyaluronan (HA) synthases and HA-binding proteins was detected parallel with an elevated presence of HA in aged PACAP KO mice. Expression of bone related collagens (I and X) was augmented in young and aged animals. These results suggest that loss of PACAP signaling results in dysregulation of cartilage matrix composition and may transform articular cartilage in a way that it becomes more prone to degenerate.

## Introduction

Articular cartilage is a unique type of connective tissue in the skeletal system with very poor regenerative capacity. Major factors behind this phenomenon are its avascular, aneural nature and the postmitotic character of adult chondrocytes. This tissue covers the articular surface of synovial joints providing frictionless gliding surface and shock absorption during locomotion (Redondo et al. 2018). High water content of the extracellular matrix (ECM) and the specific collagen fibre orientation pattern is partly responsible for the resilience, weight bearing capacity and flexibility of the tissue (Redondo et al. 2018). In the superficial layer of articular cartilage, a proliferation zone can be identified, which has an important function in maintaining the elasticity of articular surface (Antons et al. 2018). Mature articular cartilage is composed of a specific ECM with dominantly collagen type II fibres, which run in a specific arcade-like orientation, and collagen type IX and XI associate to the core of the fibres in a healthy collagen network. The ground substance of cartilage ECM is rich in proteoglycans (PG) such as aggrecan (Laasanen et al. 2003). The glycosaminoglycan hyaluronic acid (HA) is another essential component of the ECM in articular cartilage as it forms a specific network with the PGs and binds high amounts of water. In the deep layers of articular cartilage (hypertrophic zone and calcification zone), ECM becomes calcified providing a tissue with intermediate biomechanical properties interconnecting articular cartilage and subchondral bone. In this region, chondrocytes are terminally differentiated hypertrophic cells which subsequently undergo apoptosis and the whole tissue serves as a template for trabecular bone formation (Zevenbergen et al. 2018). If any of the components mentioned above are downregulated or the balance in their expression is disturbed, cartilage homeostasis can become severely impaired (Li et al. 2018). In pathological conditions, such as inflammation, oxidative stress or increased mechanical force, the composition of cartilage-specific ECM can be disintegrated and cartilage degeneration may occur (Poulet and Staines 2016).

Secretion of cartilage-specific molecules is regulated by various signaling pathways in developing articular cartilage. Activation of PKA plays a crucial role in this process (Zakany et al. 2002) and can phosphorylate Sox9 or CREB transcription factors (Zakany et al. 2005). More active, phosphorylated forms of these transcription factors are transported to the nuclei and induce the expression of aggrecan and collagen type II. Inhibition of PKA leads to decreased cartilage formation (Zakany et al. 2001; Zakany et al. 2002) primarily via the lower activation of Sox9. Besides Sox9, other members of the SoxE family can also be activated in cartilage formation such as Sox5 and Sox6 (Liu and Lefebvre 2015). Alterations in the expression of the latter two transcription factors leads to a modified, pathologically deformed skeleton and impaired cartilage formation (Lefebvre et al. 2001). Although protein kinase A (PKA) is a key regulator in cartilage formation or regeneration, several other pathways have also been shown to participate. The importance of hedgehog (HH) (Juhasz et al. 2015b), ERK (Zakany et al. 2005) or protein kinase C (PKC) signaling (Matta et al. 2011) is unquestionable. Certain members of the Ser/Thr phosphatases such as protein phosphatase 2A (PP2A) (Zakany et al. 2001) and PP2B (Zakany et al. 2005) are also proven to play a regulatory role in proper cartilage formation. Although the regulatory factors in chondroprogenitor cell differentiations are being characterized, there are still numerous signaling connections not clarified. Amongst these, pituitary adenylate cyclase activating polypeptide (PACAP) is a recently identified player.

PACAP was identified and isolated from the central nervous system (CNS) by Miyata et al. (1989). It has two biologically active forms, PACAP 1-38 and PACAP 1-27, which are evolutionarily well conserved (Vaudry et al. 2009). The neuropeptides have three major G protein coupled receptors, namely PAC1, VPAC1 and VPAC2. PAC1 receptor has the highest affinity to the neuropeptide, and the activation of the receptor triggers adenylate cyclase activity leading to increased concentration of intracellular cAMP and ultimately activates PKA signaling (Gourlet et al. 1997). A positive role of PACAP has been shown in in vitro cartilage formation (Juhasz et al. 2014a), and its protective role has been proven against oxidative stress (Juhasz et al. 2015a; Juhasz et al. 2014a) and mechanical force induced damage (Juhasz et al. 2015b; Szentleleky et al. 2019). Furthermore, PACAP receptor expression has been shown in osteoarthritic cartilage (Giunta et al. 2015), and a pivotal function of PACAP has also been demonstrated in bone formation (Jozsa et al. 2018; Juhasz et al. 2014b). Differentiation of other peripheral cells and organs can be related to proper PACAP signaling such as development of sperms (Reglodi et al. 2018c), teeth (Fulop et al. 2018; Sandor et al. 2014) and brain (Vaudry et al. 2009). On the other hand, lack of PACAP influences aging processes in different tissues (Reglodi et al. 2018b) and is related to accumulation of amyloid deposits (Reglodi et al. 2018b). Lack of the neuropeptide also modulates the vasomotor response of arteries (Ivic et al. 2017) and retina functions (Kovacs-Valasek et al. 2017) during aging.

Although there are some studies on PACAP in aging, no experimental data is available on the regulation of cartilage aging. Cells of the hyaline cartilage have extremely long lifespan but little is known about their signaling alterations in aging. As accelerated aging has been demonstrated in PACAP gene–deficient mice, we investigated the in vivo signaling alterations in young and aged wild-type (WT) and PACAP knock out (KO) mice.

## Materials and methods

### Animals

Generation and maintenance of the PACAP-deficient mice on the CD1 background have been described in detail (Hashimoto et al. 2001). They were backcrossed for at least ten generations with the CD1 strain. Genotype was tested with PCR reactions. For the experiments, we sacrificed 3-month-old (“young”) and 1-year-old (“aged”) wild-type (WT, *n* = 20–20) and homozygous PACAP-deficient (PACAP KO, *n* = 20–20) mice. Animals were fed and watered ad libitum, under light/dark cycles of 12/12 h. Hind limbs were removed after sacrificing the mice with an overdose of pentobarbital sodium (100 mg/kg bw). All procedures were performed in accordance with the ethical guidelines approved by the University of Pécs (permission number: BA02/2000-15024/2011).

### Staining procedure

Knee joints were dissected, and after additional tissues were removed, samples were washed in PBS three times and fixed in a 4:1 mixture of absolute ethanol and 40% formaldehyde. Joints were decalcified in 4% EDTA for 4 weeks until bones became soft. Then samples were dehydrated in ascending alcohol row and embedded in paraffin. Five-micrometre-thick serial sections were made. After rehydration haematoxylin-eosin (HE) (Sigma-Aldrich, MO, USA) and dimethylmethylene blue (DMMB) (Sigma-Aldrich, MO, USA), stainings were performed. All staining protocols were carried out according to the instructions of manufacturer. Photomicrographs were taken using DP74 camera (Olympus Corporation, Tokyo, Japan) on Olympus Bx53 microscope (Olympus Corporation, Tokyo, Japan).

### Cartilage thickness measurement

#### Measurement of mean thickness of cartilage on a convex segment

**Fig. 1:**
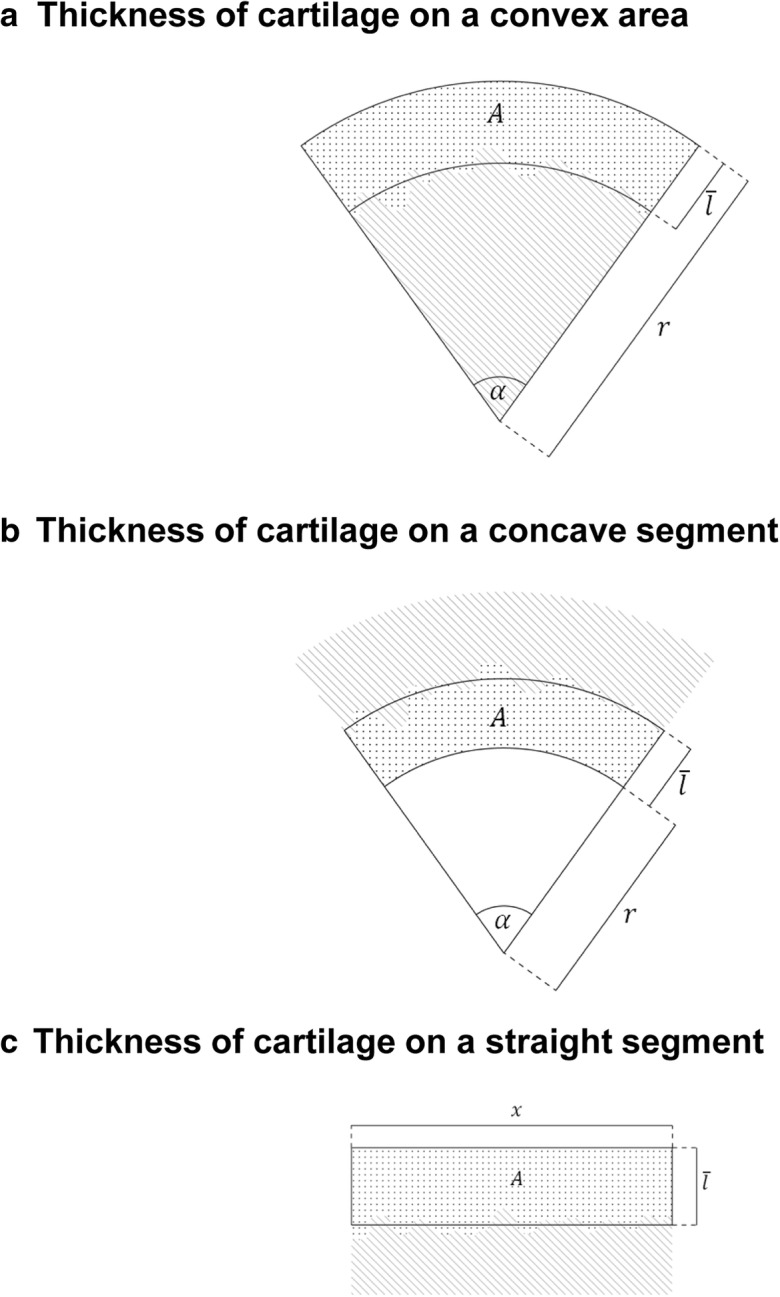
Measurement of mean thickness of cartilage. Convex segment (**a**): the mean thickness ($\overline{l}$) of the cartilage (dotted area) is the difference of the outer and inner radius of the annulus segment that has an area equal to the area (*A*) of the cartilage segment of the same *α* angle. Mean thickness can be calculated with the formula for the area of an annulus segment. The striped area represents subchondral bone. *r*—outer radius of the constructed annulus segment. Concave segment (**b**): measuring the thickness of cartilage on a concave segment. Dotted and striped areas represent cartilage and subchondral bone, respectively. *A*—area of the cartilage segment of *α*, $\overline{l}$—mean thickness, *r*—inner radius of the constructed annulus segment. Straight segment (**c**): measuring the thickness of cartilage on a straight segment. Dotted and stripped areas represent cartilage and subchondral bone, respectively. *A*—area of the cartilage segment, $\overline{l}$—mean thickness, *x*—width of the constructed rectangle and the selected segment of cartilage

For the measurement of articular cartilage thickness, a customized mathematical formula was used. A circular arc that best fits to the surface of the cartilage was constructed in *Adobe Illustrator19.0.0* (Adobe Inc., San Jose, CA, USA) with the help of the SubScribe plug-in (Astute Graphics Limited, UK). Two arbitrary perpendicular lines (radiuses, *r*) were then drawn from this arc, defining the *α* angle and the segment of the cartilage used for the measurement. Both *α* and *r* were measured in pixels in *Adobe Illustrator*. Length of the scale bar was measured as well for calculating the conversion ratio from pixels to micrometres (Fig. 1a). Area of the bound segment of the cartilage was measured using the ImageJ 1.40 g freeware freehand selection tool. Thickness ($\overline{\mathrm{l}}$) was defined as the difference of the radius of the outer and inner circles of an annulus that had an outer arc conforming to the surface of the cartilage and the area of which equals that of the portion of cartilage bound by the radiuses. Thus, *A* was the area of the annulus sector of angle *α* and was calculated as follows:

$$ A={A}_r-{A}_{r-\overline{l}} $$

$$ A={r}^2\pi \frac{\alpha }{360{}^{\circ}}-{\left(r-\overline{l}\right)}^2\pi \frac{\alpha }{360{}^{\circ}} $$

$$ 0={\overline{l}}^2\pi \frac{\alpha }{360{}^{\circ}}-2r\overline{l}\pi \frac{\alpha }{360{}^{\circ}}+A $$

$$ a=\pi \frac{\alpha }{360{}^{\circ}},\mathrm{b}=-2 r\pi \frac{\alpha }{360{}^{\circ}},c=A. $$

After rearranging and solving the quadratic equation for $\overline{l}$, the thickness of the cartilage is converted to micrometres.

#### Measurement of mean thickness of cartilage on a concave segment

Essentially, the same process was carried out, with some changes made to the equation (Fig. 1b).

$$ A={A}_{r+\overline{l}}-{A}_r $$

$$ A={\left(r+\overline{l}\right)}^2\pi \frac{\alpha }{360{}^{\circ}}-{r}^2\pi \frac{\alpha }{360{}^{\circ}} $$

$$ 0={\overline{l}}^2\pi \frac{\alpha }{360{}^{\circ}}+2r\overline{l}\pi \frac{\alpha }{360{}^{\circ}}-A $$

$$ a=\pi \frac{\alpha }{360{}^{\circ}},\mathrm{b}=2 r\pi \frac{\alpha }{360{}^{\circ}},c=-A $$

#### Measuring mean thickness of cartilage on a straight segment

Thickness ($\overline{l}$) was the height of the rectangle that had an area equal to that of and the same width (*x*) as the selected segment of cartilage (Fig. 1c).

$$ A=x\overline{l} $$

$$ \overline{l}=\frac{A}{x} $$

### Immunohistochemistry

Knee joints were dissected, and additional tissues were removed. Joints were washed in PBS three times and fixed in a 4:1 mixture of absolute ethanol and 40% formaldehyde for 24 h. Joints were decalcified in 4% EDTA for 4 weeks until bones became soft. Then, samples were dehydrated in graded ethanol series and embedded in paraffin. Five micrometres of serial sections were made. Sections were rehydrated in descending alcohol row and washed in PBST (phosphate-buffered saline supplemented with 1% Tween-20) three times. Nonspecific binding sites were blocked in PBST supplemented with 1% bovine serum albumin (BSA, Amresco LLC, Solon, OH, USA) at 37 °C for 30 min in a humidified-chamber.

For collagen typeX detection, a monoclonal anti-collagen type X (Sigma-Aldrich, MO, USA) at a dilution of 1:500 was used at 4 °C overnight. Primary antibody was visualized with anti-mouse Alexa555 secondary antibody (Life Technologies Corporation, Carlsbad, CA, USA) at a dilution of 1:1000. Slides were mounted in Vectashield Hard Set mounting medium (Vector Laboratories, Peterborough, England).

Hyaluronic acid (HA) was detected by using a biotinylated HA-binding complex in 5 μg/mL concentration (bHABC, kindly provided by R. Tammi and M. Tammi, Department of Anatomy, University of Kuopio, Kuopio, Finland) at 4 °C overnight. The reaction was visualized with Streptavidin-Alexa555 (2 μg/mL, Invitrogen Corporation, Carlsbad, CA, USA) for fluorescence microscopy. Tissues were mounted in Vectashield Hard Set mounting medium (Vector Laboratories, Ltd. Peterborough, UK).

Photomicrographs of collagen type X immunohistochemistry were taken using an Olympus DP72 camera on a Nikon Eclipse E800 microscope (Nikon Corporation, Tokyo, Japan). Images were acquired using cellSense Entry 1.5 software (Olympus, Shinjuku, Tokyo, Japan) with constant camera settings to allow comparison of fluorescent signal intensities. Images of Alexa555 were overlaid using Adobe Photoshop version 10.0 software.

### RT-PCR analysis

Articular cartilage from knee joints was isolated under microscope, and samples were kept in RNAlater (Sigma-Aldrich MO, USA). Tissues were cryo-ground in liquid nitrogen and dissolved in Trizol (Applied Biosystems, Foster City, CA, USA) and kept at − 70 °C for 24 h, and after the addition of 20% RNase-free chloroform, samples were centrifuged at 4 °C at 10,000×*g* for 15 min. Samples were incubated in 500 μL of RNase-free isopropanol at − 20 °C for 1 h; then, total RNA was harvested in RNase free water and stored at − 20 °C. The assay mixture for reverse transcriptase reaction contained 2 μg RNA, 0.112 μM oligo(dT), 0.5 mM dNTP and 200 units of High Capacity RT (Applied Bio-Systems, Foster City, CA, USA) in 1× RT buffer. For the sequences of primer pairs and further details of polymerase chain reactions, see Table 1. Amplifications were performed in a thermal cycler (Labnet MultiGene™ 96-well Gradient Thermal Cycler; Labnet International, Edison, NJ, USA) in a final volume of 11 μL (containing 1 μL forward and reverse primers [0.4 μM], 0.5 μL dNTP [200 μM], and 5 units of Promega GoTaq® DNA polymerase in 1× reaction buffer) as follows: 95 °C, 2 min, followed by 35 cycles (denaturation, 94 °C, 1 min; annealing at optimized temperatures as given in Table 1 for 1 min; extension, 72 °C, 90 s) and then 72 °C, 10 min. PCR products were analysed by electrophoresis in 1.2% agarose gel containing ethidium bromide. Actin was used as internal control. Optical density of signals was measured by using ImageJ 1.40 g freeware, and results were normalized to the optical density of control tissue.

**Table 1:** Nucleotide sequences, amplification sites, GenBank accession numbers, amplimer sizes and PCR reaction conditions for each primer pair are shown

Gene	Primer	Nucleotide sequence (5′ → 3′)	GenBank ID	Annealing temperature (°C)	Amplimer size (bp)
preproPACAP	Sense	GAA GAC GAG GCT TAC GAC CA (314–333)	NM_001315503.1	58	288
	Antisense	GTC CGA GTG GCG TTT GGT (584–601)			
PAC1	Sense	TATTACTACCTGTCGGTGAAG(912–932)	NM_007407.4	52	213
	Antisense	ATGACTGCTGTCCTGCTC (1107–1124)			
VPAC1	Sense	TTT GAG GAT TTC GGG TGC (974–991)	NM_011703.4	53	266
	Antisense	TGG GCC TTA AAG TTG TCG (1222–1239)			
VPAC2	Sense	CTC CTG GTA GCC ATC CTT (805–822)	NM_009511.2	53	149
	Antisense	ATG CTG TGG TCG TTT GTG (936–953)			
PKA (Prkaca)	Sense	GCAAAGGCTACAACAAGGC (847–865)	NM_008854	53	280
	Antisense	ATGGCAATCCAGTCAATCG (1109–1126)			
CREB (Creb1)	Sense	AGATTGCCACATTAGCCC (95–112)	*NM_031017.1*	52	441
	Antisense	GCTGTATTGCTCCTCCCT (518–535)			
Sox9	Sense	GTA CCC GCA TCT GCA CAA CG (378–397)	NM_011448	62	521
	Antisense	GTG GCA AGT ATT GGT CAA ACT CAT T (874–898)			
Sox6	Sense	GGA GTC GGG AGC GTG AAA (483–500)	NM_001277326.1	54	381
	Antisense	GGC GAG CAA GGT CCA TTT (846–863)			
Sox5	Sense	GCT CCA TAC AAC TCA TCT AC (504–523)	NM_011444.3	53	181
	Antisense	TGT CTT CTG GCT CAT TCT (667–684)			
Aggrecan	Sense	CGG GAA GGT TGC TAT GGT G (782–800)	NM_007424.2	59	359
	Antisense	CCT GTC TGG TTG GCG TGT A (1122–1140)			
Col2a1	Sense	AAA GAC GGT GAG ACG GGA GC (1900–1919)	NM_001113515	63	289
	Antisense	GAC CAT CAG TAC CAG GAG TGC C (2167–2188)			
Chst11	Sense	TGC TAT GTG CCC AAG GTA (822–839)	NM_021439.2	55	466
	Antisense	CGA GGT CGT AGT GGA TGTG (1269–1287)			
HAS2	Sense	ACAGGCATCTCACGAACC (1479–1496)	NM_008216.3	51	415
	Antisense	ATC TTG GCG GGA AGT AAA (1876–2893)			
HAS3	Sense	TCGGCGATTCGGTGGACT (835–852)	NM_001331048.1	59	280
	Antisense	TGCTGGAGGAGGCTGTTGC (1096–1114)			
HAPLN1	Sense	GGC TCA GGA ATC CAC AAA (217–234)	BC066853	55	284
	Antisense	GGA AAG TAA GGG AAC ACC A (482–500)			
RHAMM	Sense	GAG GGA CTC AGG ACA AAC (374–391)	NM_013552.2	49	485
	Antisense	TTC TTC TAA CTG GGC AAT (8412–858)			
CD44	Sense	GGATTCATCCCAACGCTAT (600–618)	NM_009851.2	53	216
	Antisense	ACT CGC CCT TCT TGC TGT (798–815)			
Col1a1	Sense	GGG CGA GTG CTG TGC TTT (237–254)	BC050014	62	388
	Antisense	GGG ACC CAT TGG ACC TGA A (606–624)			
Col10a1	Sense	TTC TGG GAT GCC GCT TGT C (1602–1620)	NM_009925	61	263
	Antisense	TCG TAG GCG TGC CGT TCT T (1846–1864)			
Actin (Actb)	Sense	GCCAACCGTGAAAAGATGA (419–437)	*NM_007393.5*	54	462
	Antisense	CAAGAAGGAAGGCTGGAAAA (861–880)			

### Western blot analysis

Articular cartilage of knee joints was removed under microscope and was kept in 100 μL of homogenization RIPA. Tissues were cryo-ground in liquid nitrogen. After centrifugation, tissue pellets were suspended in 100 μL of homogenization RIPA and samples were stored at − 70 °C. Suspensions were sonicated by pulsing burst for 30 s at 40 A (Cole-Parmer, IL, USA). For setting equal protein concentration, BCA protein assay was used (Pierce™, MA, USA) and Laemmli electrophoresis sample buffer (4% SDS, 10% 2-mercaptoethanol, 20% glycerol, 0.004% bromophenol blue, 0.125 M TrisHCl pH 6.8) was added to cell lysates and boiled for 10 min. About 10 μg of protein was separated by 7.5% SDS-PAGE gel for detection of PAC1, VPAC1, VPAC2, PKA, CREB, P-CREB, Sox9, P-Sox9, Sox5, Sox6, collagen type I, collagen type X, Chst11, HAS2, HAS3, RHAMM, HAPLN1, and CD44 and by 5% SDS-PAGE gel for detection collagen type II and aggrecan. Proteins were transferred electrophoretically to nitrocellulose membranes with TransTurbo blot (Bio-Rad Laboratories, CA, USA) in 10 min. After blocking with 5% non-fat dry milk in phosphate-buffered saline (PBST) with 0.1% Tween 20, membranes were washed and exposed to the primary antibodies overnight at 4 °C in the dilution as given in Table 2. After washing for 30 min in PBST, membranes were incubated with anti-rabbit IgG (Bio-Rad Laboratories, CA, USA) in 1:1500 and anti-mouse IgG (Bio-Rad Laboratories, CA, USA) in 1:1500 dilution. Signals were detected by enhanced chemiluminescence (Advansta Inc., San Jose, CA, USA) according to the instructions of the manufacturer and documented by gel documentary system (Fluorchem E, ProteinSimple, CA, USA). Optical density of Western blot signals was measured by using ImageJ 1.40g freeware, and results were normalized to that of control samples.

**Table 2:** Table of antibodies used in the experiments

Antibody	Host animal	Dilution	Distributor
Anti-PAC1	Rabbit, polyclonal	1:600	Sigma-Aldrich, St. Louis, MO, USA
Anti-VPAC1	Rabbit, polyclonal	1:800	Alomone Labs., Jerusalem, Israel
Anti-VPAC2	Rabbit, polyclonal	1:600	Abcam, Cambridge, UK
Anti-Coll. I.	Mouse, monoclonal	1:1000	Sigma-Aldrich, St. Louis, MO, USA
Anti-CREB	Rabbit, polyclonal	1:800	Millipore, Billerica, MA, USA
Anti-P-CREB	Rabbit, polyclonal	1:800	Millipore, Billerica, MA, USA
Anti-Coll. II.	Mouse, monoclonal	1:500	Novus Biologicals, Littleton, CO, USA
Anti-HAS2	Rabbit, polyclonal	1:300	Santa Cruz Biotechnology Inc., Santa Cruz, CA, USA
Anti-HAS3	Rabbit, polyclonal	1:300	Sigma-Aldrich, St. Louis, MO, USA
Anti-Coll. X.	Rabbit, polyclonal	1:500	Sigma-Aldrich, St. Louis, MO, USA
Anti-Sox9	Rabbit, polyclonal	1:600	Abcam, Cambridge, UK
Anti-P-Sox9	Rabbit, polyclonal	1:800	Sigma-Aldrich, St. Louis, MO, USA
Anti-PKA	Rabbit, polyclonal	1:800	Cell Signaling, Danvers, MA, USA
Anti-RHAMM	Mouse, monoclonal	1:500	Novocastra Laboratories Ltd., Newcastle, UK
Anti-Aggrecan	Rabbit, polyclonal	1:600	Millipore, Billerica, MA, USA
Anti-HAPLN1	Mouse, monoclonal	1:500	R&D Systems, Minneapolis, MN, USA
Anti-Chst11	Rabbit, polyclonal	1:600	Sigma-Aldrich, St. Louis, MO, USA
Anti-CD44	Mouse, polyclonal	1:800	R&D Systems, Minneapolis, MN, USA
Anti-Sox5	Rabbit, polyclonal	1:500	Abcam, Cambridge, UK
Anti-Sox6	Rabbit, polyclonal	1:500	Abcam, Cambridge, UK
Anti-actin	Mouse, monoclonal	1:10000	Sigma-Aldrich, St. Louis, MO, USA

### Statistical analysis

All data are representative of at least five independent experiments. Where applicable, data are expressed as mean ± SEM. Statistical analysis was performed by Student’s *t* test. Threshold for statistically significant differences as compared to respective control (wild-type animals) was set at *p < 0.05.

## Results

### Thickness of articular cartilage increased in aged PACAP gene–deficient mice

**Fig. 2:**
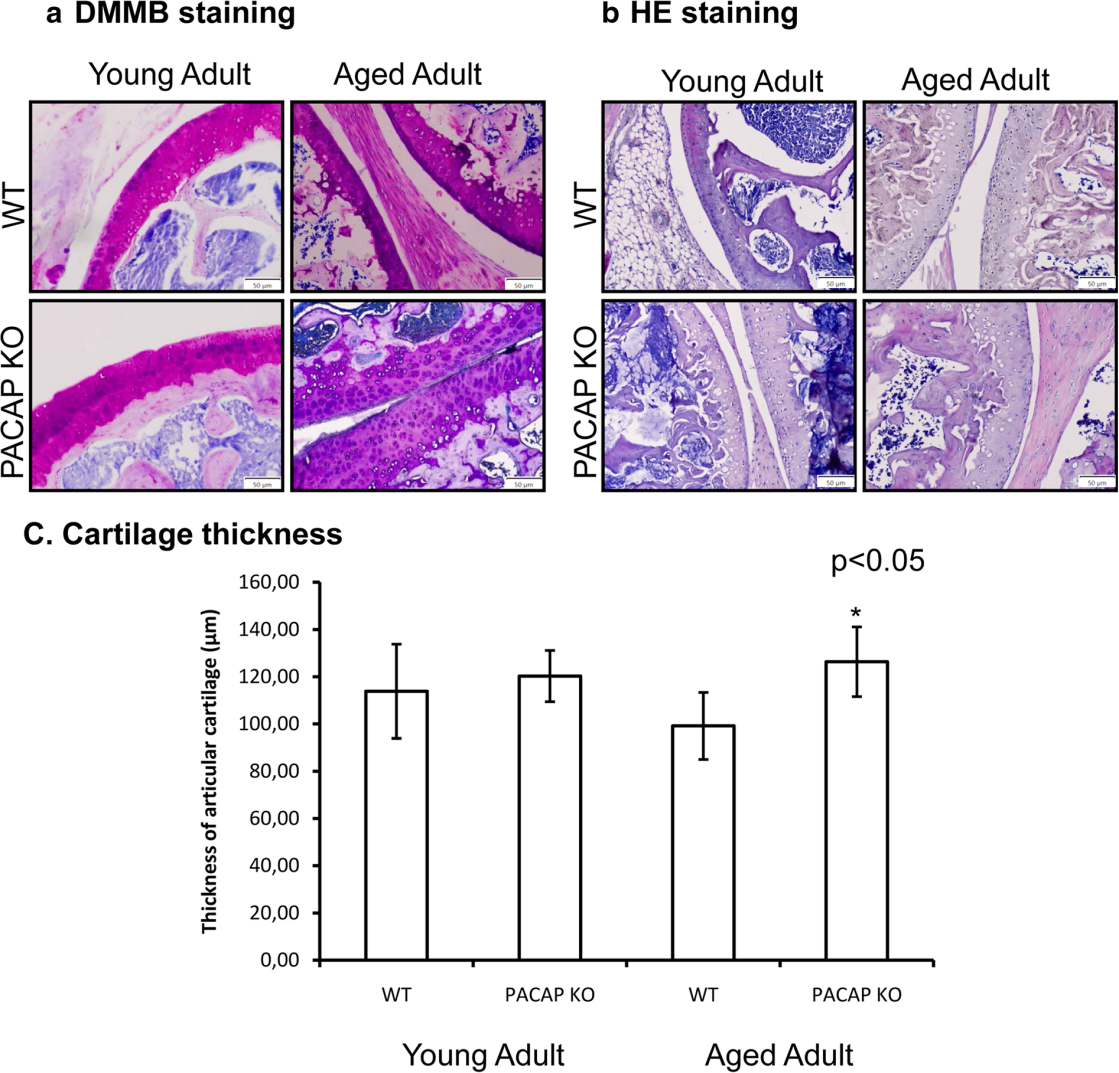
Morphological analysis of knee joints of young and aged WT and PACAP KO mice. Dimethylmethylene blue (DMMB) (**a**) and hematoxylin-eosin (HE) staining (**b**) were used to visualize the histological differences. Original magnification was × 20. Scale bar: 50 μm. Geometric analysis (**c**) of mouse articular cartilage. Representative data of 10 independent experiments. Asterisks indicate significant (*p < 0.05) difference in thickness of cartilage compared to the respective control

DMMB staining was performed to demonstrate the presence of metachromatic ECM components (PGs, sulphated GAGs). There was similar metachromasia in young WT and PACAP gene–deficient mice, while metachromasia was paler in aged KO cartilage (Fig. 2a). No significant macroscopic alterations were detected in the morphology of young WT or PACAP KO articular cartilage with HE staining. Furthermore, morphologically, no differences were visible in the articular cartilage of knee joints in aged WT or PACAP gene–deficient mice (Fig. 2b). Thickness of articular cartilage was measured with a mathematical geometric method in 10 different joints both in tibial and femoral articular cartilages. In young PACAP animals, cartilage was tendentiously thicker but no significant alterations were measured compared with WT mice (Fig. 2c). On the contrary, significantly thicker cartilage was detected in aged PACAP KO mice compared with aged WT animals (Fig. 2c).

### PACAP receptors diminished in aged cartilage of PACAP KO mice

**Fig. 3:**
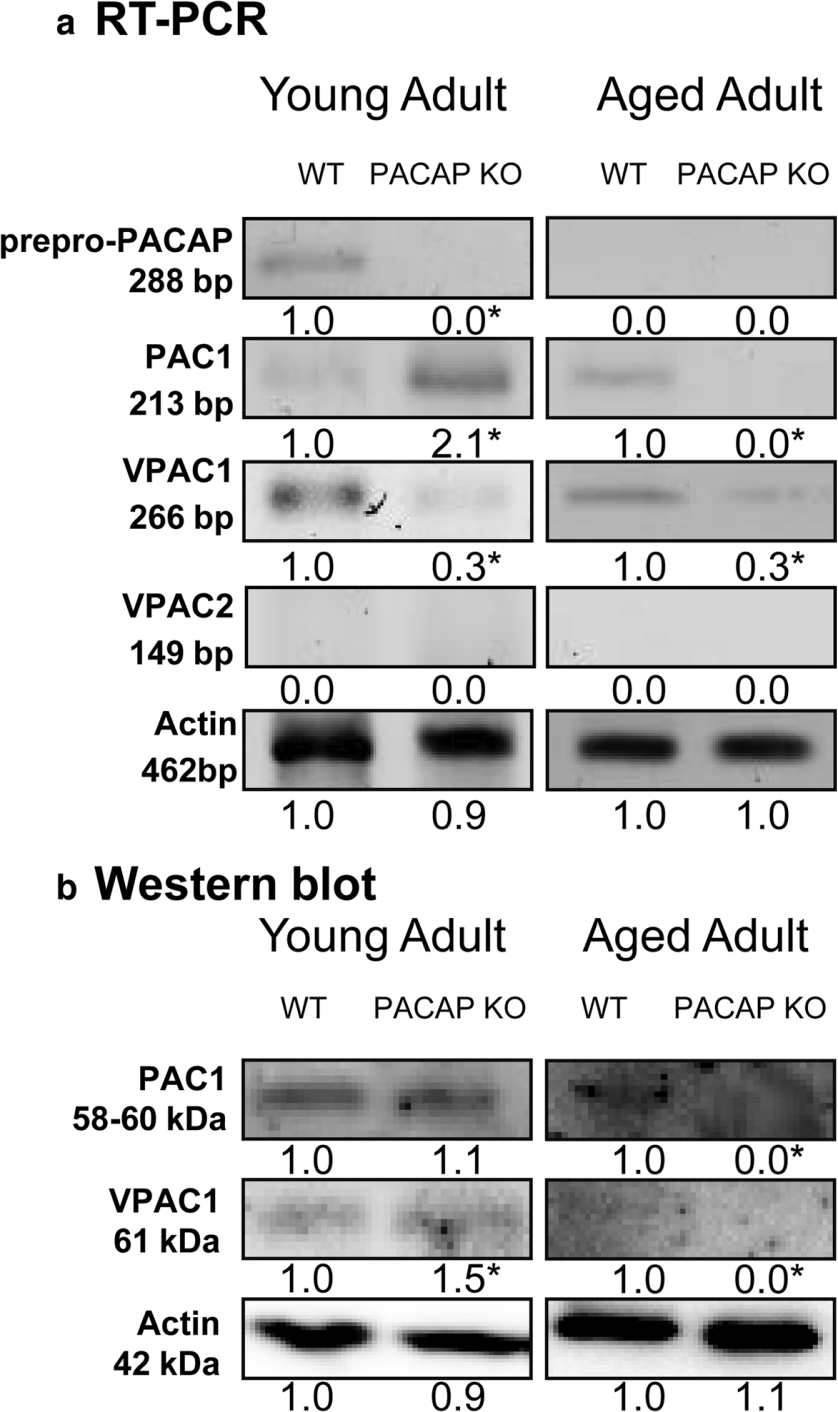
Investigation of PACAP and its receptors in articular cartilage. mRNA (**a**) and protein (**b**) expression of preproPACAP, PAC1, VPAC1 and VPAC2 receptors of cartilage. For RT-PCR and Western blot reactions, actin was used as control. Optical signal density was measured and results were normalized to the WT controls. For **a** and **b**, numbers below signals represent integrated signal densities determined by ImageJ software. Asterisks indicate significant (*p < 0.05) alteration of expression compared to the respective control. Representative data of 5 independent experiments

As PACAP can act on three different receptors, we monitored mRNA and protein expressions of PAC1, VPAC1 and VPAC2 receptors in articular cartilage. In young animals, only WT cartilage showed PACAP expression as it was expected (Fig. 3a). On the other hand, no preproPACAP expression was detected in aged WT or PACAP KO animals (Fig. 3a). Expression of the main PACAP-binding receptor, PAC1, was detected in WT and PACAP gene–deficient young animals with equal expression (Fig. 3a, b). In aged cartilage, no PAC1 receptor mRNA or protein expression was detected (Fig. 3a, b). Moreover, VPAC1 expression was similar to PAC1 with an absence in aged PACAP gene–deficient mice (Fig. 3a, b), while VPAC2 was not detectable in hyaline cartilage.

### Expression of canonical PACAP downstream signaling molecules altered in PACAP gene–deficient animals

**Fig. 4:**
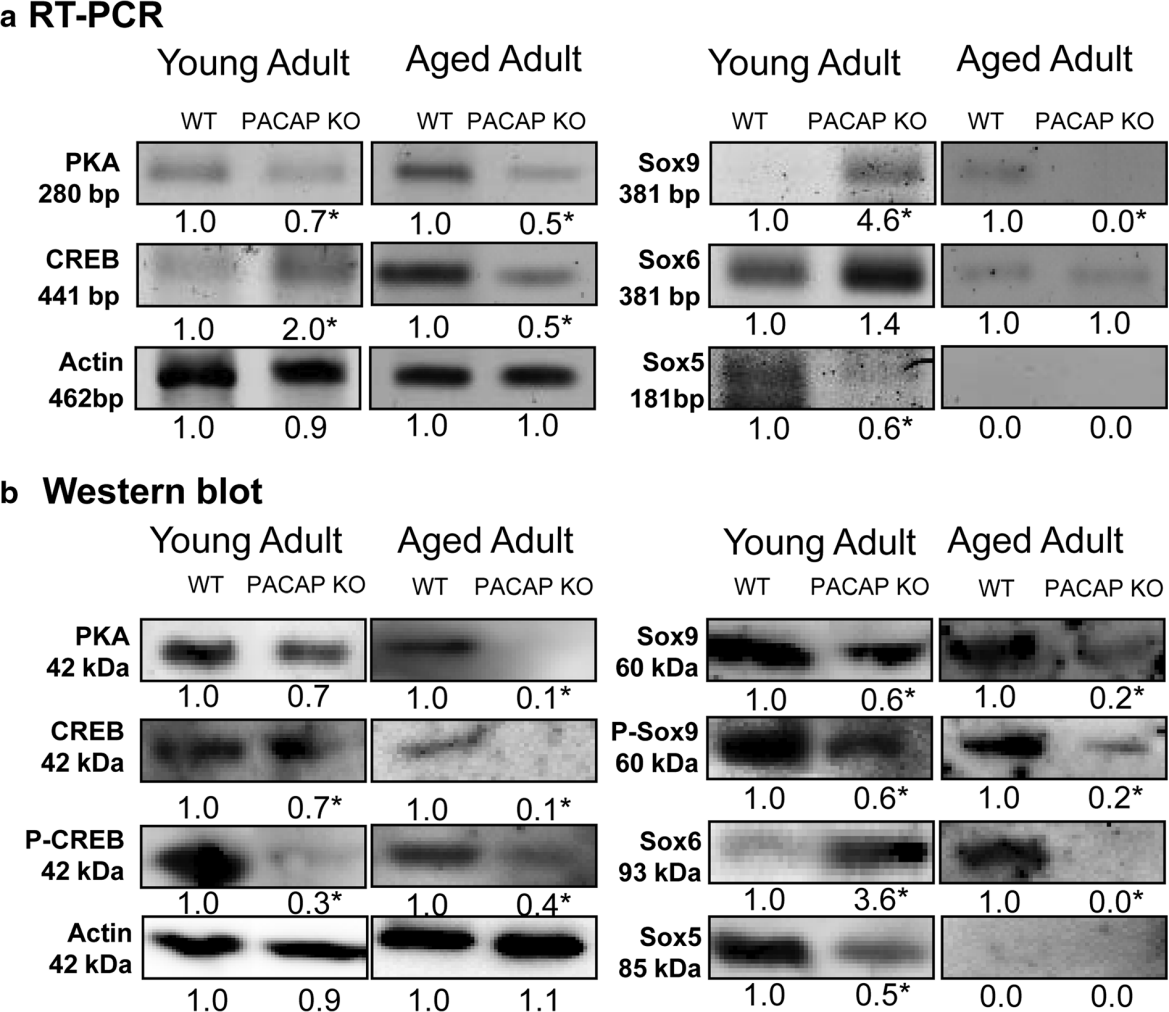
Investigation of canonical PACAP signalization in cartilage. mRNA (**a**) and protein (**b**) expression of PKA, CREB, Sox9, Sox6, and Sox5. For RT-PCR and Western blot reactions, actin was used as control. Optical signal density was measured and results were normalized to the WT controls. For **a** and **b**, numbers below signals represent integrated signal densities determined by ImageJ software. Asterisks indicate significant (*p < 0.05) alteration of expression compared to the respective control. Representative data of 5 independent experiments

Next, classical downstream targets of PACAP binding were investigated. Both the mRNA and protein expressions of PKA were reduced in young PACAP KO cartilages (Fig. 4a, b). Moreover, in aged PACAP, gene–deficient cartilage protein expression of PKA was hardly detectable with this method (Fig. 4a, b). CREB mRNA and protein expressions did not significantly change in young PACAP KO articular cartilage but its phosphorylated, more active form decreased in aged cartilage. The mRNA and protein expressions of CREB and the protein expression of P-CREB were reduced (Fig. 4a, b). Next, expression of SoxE family members followed. In young cartilage, the mRNA expression of Sox9 was significantly elevated, while a significant decrease was detected in Sox9 and P-Sox9 protein expressions (Fig. 4a, b). Analysis of Sox6 expression showed an unexpected result. In young PACAP KO animals elevated mRNA and protein expressions of Sox6 were detected (Fig. 4a, b), while Sox6 protein became undetectable in aged PACAP gene–deficient mice (Fig. 4a, b). Finally, Sox5 mRNA and protein expressions significantly decreased in young PACAP KO cartilage and no expression was detected in old WT and PACAP KO mice (Fig. 4a, b).

### Alterations of ECM production in PACAP KO mice

**Fig. 5:**
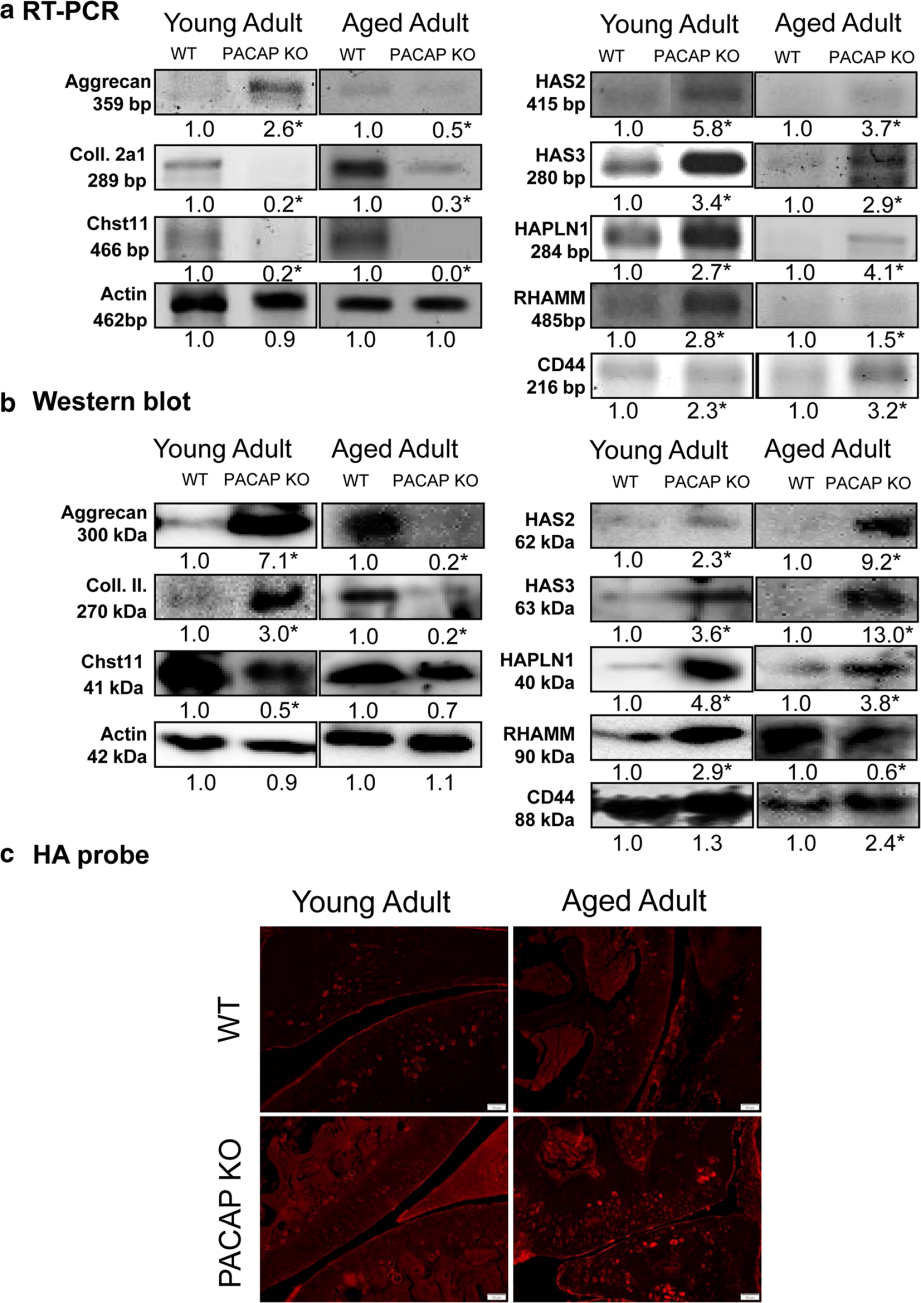
ECM production in articular cartilage. **a** mRNA and **b** protein expressions of Aggrecane, collagen type II, HAPLN1, HAS2, HAS3, RHAMM and CD44 in articular cartilage. Actin was used as a control. Numbers below signals represent integrated signal densities determined by ImageJ software. Asterisks indicate significant (*p < 0.05) alteration of expression compared to the respective control. **c** HA-binding probe in hyaline cartilage. Original magnification was × 20. Scale bar: 50 μm. Representative data of 5 independent experiments

In further steps, effects of PACAP and aging were monitored in production of cartilage specific ECM. Aggrecan, the main proteoglycan of hyaline cartilage showed an elevated mRNA and protein expression in young PACAP gene–deficient mice (Fig. 5a, b). On the contrary, both the mRNA and protein expressions were reduced in aged PACAP KO mice (Fig. 5a, b). Interestingly, the mRNA expression of collagen type II was diminished, but increased protein expression was detected in young PACAP KO cartilage (Fig. 5a, b). In aged PACAP gene–deficient mice, both the mRNA and protein expressions of collagen type II were decreased (Fig. 5a, b). Chst11 catalyses the transfer of sulphate to chondroitin. Both the mRNA and protein expressions of this transferase decreased in young and aged PACAP gene–deficient articular cartilage (Fig. 5a, b). HAPLN1 (link protein) can stabilize the aggregates of aggrecan monomers with HA and helps with the formation of the cartilage specific ECM network. Similarly to aggrecan core, protein expression HAPLN1 showed an increased expression in young PACAP KO animals but it reduced during aging (Fig. 5a, b). Production of HA in cartilage is regulated by two hyaluronan synthases, HAS2 and HAS3. Interestingly, the mRNA and the protein expressions of these enzymes elevated in both young and aged PACAP KO mice (Fig. 5a, b). Binding of HA can occur intracellularly by RHAMM or on the cell surface by CD44. mRNA and protein expressions of RHAMM and CD44 were elevated in young PACAP gene–deficient mice (Fig. 5a, b). On the contrary, RHAMM expression decreased in aged PACAP KO mice. CD44 protein expression similarly to HAS enzymes elevated in aged PACAP gene–deficient animals (Fig. 5a, b).

HA content of articular cartilage was also investigated, and high amount of GAG was visible around the chondrons of WT articular cartilage (Fig. 5c). The amount of HA was increased both in young and aged PACAP gene–deficient mice (Fig. 5c).

### Bone-specific collagens accumulated in PACAP KO cartilage in aging

**Fig. 6:**
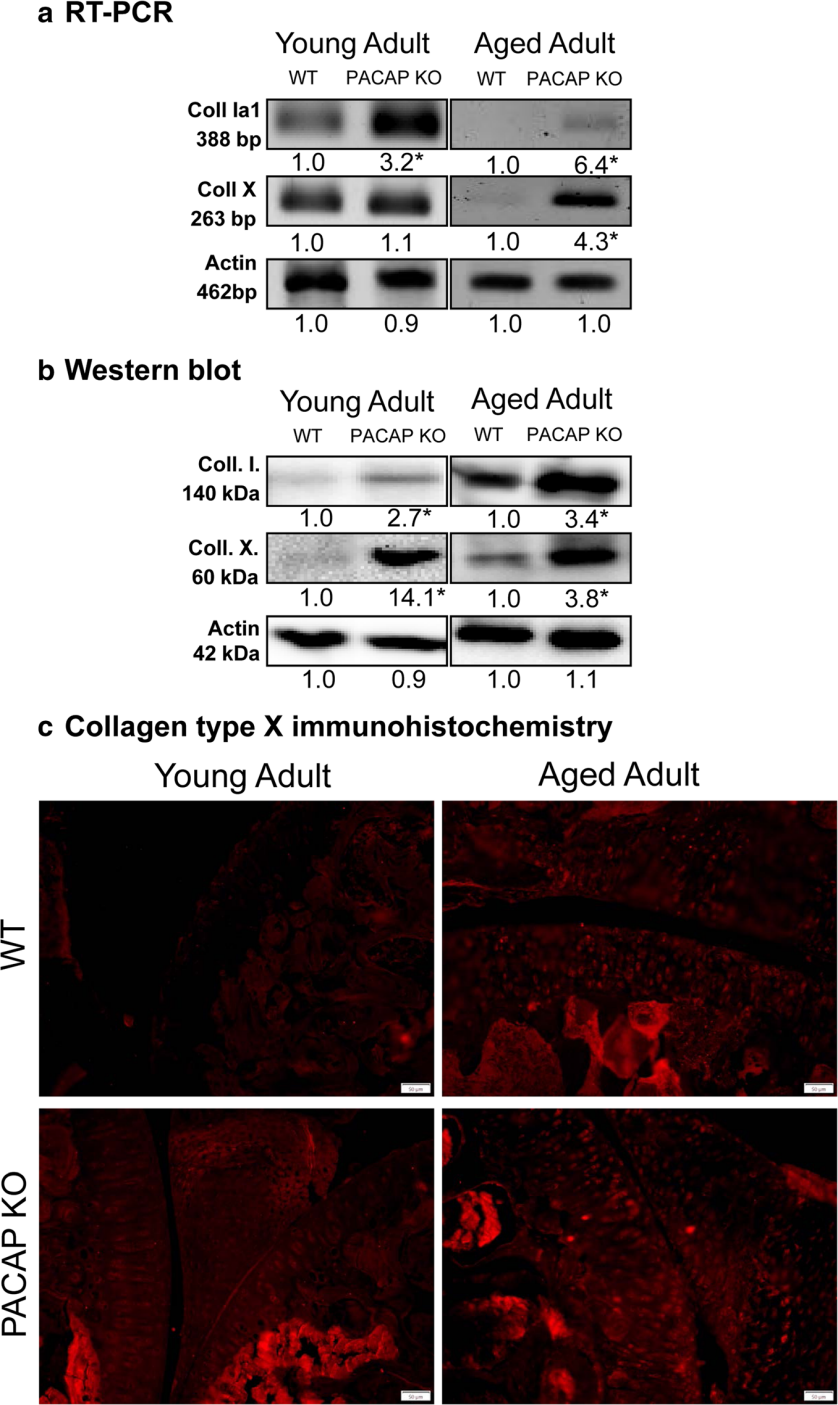
Collagen expression in cartilage. mRNA (**a**) and protein (**b**) expressions of collagen type I and X in cartilage. For RT-PCR and Western blot reactions, actin was used as controls. Numbers below signals represent integrated signal densities determined by ImageJ software. Asterisks indicate significant (*p < 0.05) alteration of expression compared to the respective control. **c** Collagen type X immunohistochemistry in hyaline cartilage. Original magnification was × 20. Scale bar: 50 μm. Representative data of 5 independent experiments

Furthermore, we investigated the expression of collagen type I and X which specifically appears in bony calcified structures. mRNA and protein expressions of collagen type I increased in young PACAP KO cartilage, and even stronger elevation was detected in aged PACAP KO joints (Fig. 6a, b). Collagen type X mRNA expression did not significantly alter, but dramatic protein expression elevation was detected in young PACAP KO mice (Fig. 6a, b). In aged PACAP KO joints, highly elevated mRNA and protein expression of collagen type X were detected (Fig. 6a, b). Location of collagen type X was followed by immunohistochemistry. In young WT mice, almost undetectable amount of collagen type X was shown in articular cartilage, while subchondral bone showed normal immunopositivity (Fig. 6c). In PACAP KO young animals, intensified immunopositivity was identified in subchondral bone and increased positivity was visible in articular cartilage (Fig. 6c). Both in WT and PACAP KO–aged mice, strong signal was detected in the subchondral region; moreover, strong collagen type X positivity appeared around the chondrons of articular cartilage (Fig. 6c).

## Discussion

In the present work, we demonstrated that genetical ablation of PACAP causes morphological and biochemical alterations in articular cartilage possibly turning this tissue more prone to degenerative diseases such as osteoarthritis (OA). Mature articular cartilage is an avascular and aneural tissue with a very low metabolic activity. Chondrocytes live in a milieu where they are exposed to acidic pH and low oxygen tension, and nutrients are transported to the cells by diffusion from the synovial fluid. Developing chondrocytes establish the specific zonal architecture of articular cartilage during foetal development (Zhang et al. 2012). Terminally differentiated hypertrophic chondrocytes secrete a cartilage matrix rich in collagen type X and the calcified cartilage of hypertrophic zone in articular cartilage provides a biomechanical transition between hyaline cartilage and the subchondral bone (Mora et al. 2018). Biologically proper internal structure of articular cartilage is needed for the optimal weight bearing capability and joint function. Several signaling pathways contribute to the formation of collagen type II-rich ECM (Fodor et al. 2013; Juhasz et al. 2014c; Matta et al. 2008; Matta et al. 2011; Varga et al. 2011; Zakany et al. 2005; Zakany et al. 2002). In aging, the normal homeostasis of articular cartilage becomes shifted toward matrix degradation. Age related alterations of chondrocytes phenotype and signaling or remodeling disorders have been reviewed by Davies et al. (2017). Injury or overload of articular cartilage, particularly in elderly people, can induce OA (Bolduc et al. 2018) and when it developes, a progressive degradation of ECM may lead to a complete destruction of cartilage (Jimenez et al. 2018; Mora et al. 2018). Aging or OA-related matrix degradation of articular cartilage is hardly treatable; only few methods are used in the clinical practice such as advanced glycation end products (AGE) treatment (Satake et al. 2018) or corticosteroid injections (Mora et al. 2018). With these interventions, decrease of joint inflammation and conservation of the status of articular cartilage can be the main goal. Shifting aging-related cartilage alterations toward later life periods or modifying senescent chondrocytes can be another possibility (Hou et al. 2018; Jeon et al. 2018). Currently, stem cell therapy seems an emerging method for prevention both of the age-related cartilage degradation and OA formation (Krajewska-Wlodarczyk et al. 2018).

PACAP was found to exert protecting effects in different tissues. It can be beneficial in treatments of retinopathy (Szabadfi et al. 2016), Parkinson’s disease (Brown et al. 2014) and ischemia (Reglodi et al. 2018d). It is also known that PACAP induces the activation of anti-apoptotic processes and leads to decreases of pro-apoptotic factors (Reglodi et al. 2018a). Deficient PACAP-signaling was found in various neurodegenerative diseases such as Alzheimer’s disease (Wu et al. 2006) and Parkinson’s disease (Feher et al. 2018). Furthermore, reduced metabolism is detected in aging process (LaRoche et al. 2018; Nacarelli et al. 2018; Tian et al. 2017) which can be compensated by PACAP (Mansouri et al. 2016). Aging can lead to several skeletal and oral diseases such as dental root caries or alveolar bone loss (An et al. 2018); on the other hand, PACAP regulates the development of teeth (Fulop et al. 2018). Regarding cartilage and bone development and disease models, our group previously described that PACAP has a positive effect on chondrogenesis with the elevation of Sox9 activation (Juhasz et al. 2014a) and prevents the harmful effects of oxidative stress and mechanical overload in in vitro chondrogenesis (Juhasz et al. 2014a; Juhasz et al. 2015b). The presence of PACAP receptors in hyaline cartilage was demonstrated in chicken high density chondrifying cell cultures (Juhasz et al. 2014a) and in human OA (Giunta et al. 2015). Furthermore, the addition of PACAP influenced the expression of ECM molecules, such as HA (Juhasz et al. 2014a) or collagens (Juhasz et al. 2015b). The lack of PACAP resulted in a signaling disorder femur development (Jozsa et al. 2018). Although the importance of PACAP-signaling and its positive role in OA is likely (Grassel and Muschter 2018), the role of PACAP in pathogenesis of cartilage disorders is not extensively studied and no information is available about its function in cartilage aging.

It is known that increased thickness of calcified cartilage can be detected in hip joints in early stages of OA (Hartlev et al. 2018). In knee joints of aged PACAP KO mice, significant thickening of articular cartilage and subchondral bone, along with decreased metachromasia, was our most obvious histological observation. This increase was parallel with disturbed ratio of the various components in the ECM. In aged PACAP KO animals, more collagen type I, elevated HA content was detected along with decrease of aggrecan and type II collagen. Loss of aggrecan was also proved by reduced metachromasia with DMMB. Altogether, these alterations can lead to softening of the articular cartilage which could induce thickening of subchondral bone. These microscopic morphological changes seem very similar to initial stages of OA (Lahm et al. 2017). It also suggests that the neuropeptide is involved in the inhibition of articular cartilage aging by maintaining chondrocyte plasticity (Varela-Eirin et al. 2018).

**Fig. 7:**
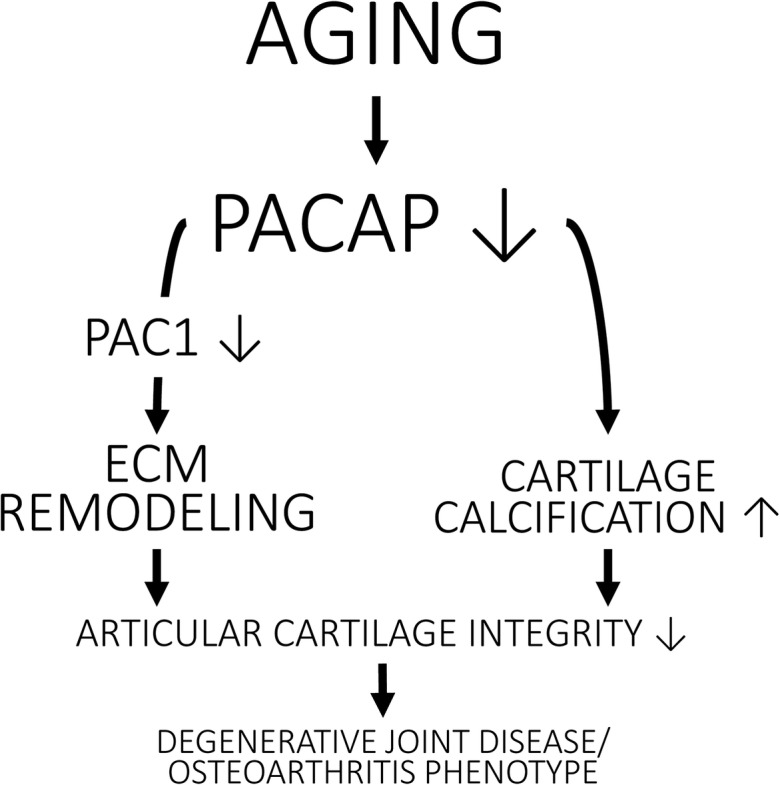
PACAP function in aging. Concentration of PACAP and expression of PAC1 receptor reduce in aging process of articular cartilage. Reduction of the neuropeptide induces decreased cartilage remodeling but enhances the calcification processes of articular cartilage. Therefore, PACAP is important in maintaining cartilage integrity and prevents formation of pathological illnesses such as osteoarthritis

Along with aging, loss of PAC1 receptor was detected in articular cartilage of PACAP KO mice, which can be a factor triggering an ECM formation disorder (Fig. 7). This finding further supports the importance of PAC1 receptor activation in cartilage differentiation and proper ECM synthesis (Juhasz et al. 2014a). Similarly, PAC1 receptor reduction was also shown in retinal aging of PACAP KO mice (Kovacs-Valasek et al. 2017), although elevation was detected in young gene deficient mice. This further strengthens the hypothesis that presence of PACAP receptors exerts tissue-specific effects during aging (Reglodi et al. 2018b). VPAC1 receptor expression was identified in articular cartilage, its expression was almost undetectably low, suggesting that PAC1 is the dominant receptor in this tissue (Juhasz et al. 2014a).

Although the expression of PKA is not PACAP-dependent, we detected reduced PKA expression in aged PACAP gene–deficient mice. As PKA phosphorylates CREB and Sox9 transcription factors, this observation can explain the decreased activation and expression of these transcription factors. Reduced phosphorylation of CREB and Sox9 was more pronounced in aged articular cartilage (Zhang et al. 2016). We have described earlier that activation of Sox9 also depends on PAC1 receptor activation (Juhasz et al. 2014a), and the lack of the neuropeptide further decreased the Sox9-dependent aggrecan and collagen expression (Zhang et al. 2016). These results suggest that the presence of PACAP is necessary for maintenance of continuous activation of Sox9 to produce and maintain the cartilage specific matrix expression. Sox5 and Sox6 are also positive regulators of chondrogenesis (Nishimura et al. 2018), and the lack of PACAP reduces the expression of Sox5 but elevates Sox6 expression. It seems that the reduced Sox9 expression is compensated in the lack of the neuropeptide by the elevated Sox6 expression in articular cartilage of young adults. A very similar phenomenon was identified in testis of PACAP KO mice, where the decreased Sox9 expression was compensated by an elevated Sox10 production (Reglodi et al. 2018c). Sox6 elevation resulted in the increase of aggrecan and collagen type II expression in young PACAP gene–deficient mice, accounting for the normal cartilage morphology. Similar compensatory mechanisms were identified in teeth of PACAP KO mice by the activation of Notch signaling (Fulop et al. 2018) or in femurs where augmented BMP signaling normalized the bone formation (Jozsa et al. 2018). Sox5 and Sox6 play important roles in the formation of proper articular cartilage morphology during development (Dy et al. 2010), but Sox5 function is diminished in aging process. Although Sox6 is detectable in aged WT mice, it is not present in PACAP KO animals. The decreased expression of Sox6 resulted in the loss of compensatory mechanism and further reduced aggrecan and collagen type II expression leading to the structural disorder of ECM in articular cartilage of PACAP gene–deficient mice. Moreover, lowered transferase (Chst11) expression can be the explanation of the decreased presence of sulphated glycosaminoglycans which was shown by a paler metachromasia in aged PACAP KO mice. Sox9, Sox5 and Sox6 are the major transcription factors of chondrogenesis, and their activation results various gene activations (Akiyama and Lefebvre 2011). Here we gave evidence that PACAP-signaling plays an essential role in Sox9 and Sox5 function, but Sox6, at least, partly was PACAP independently activated and compensated the lowered function of Sox9 and Sox5 in young PACAP KO mice. Our results suggest that the activation of Sox transcription factors is age dependent and in case of Sox9 this was demonstrated previously (Zhang et al. 2016). We observed that the absence of PACAP further reduced Sox9 and Sox5 function, and it further strengthens the hypothesis that PACAP can keep balancing in crosstalks of various signaling pathways even during aging of tissues (Fig. 7) (Jozsa et al. 2018; Niewiadomski et al. 2013; Szentleleky et al. 2019).

Interestingly, the thickness of articular cartilage increases in aging PACAP KO mice; therefore, we analysed the HA homeostasis of hyaline cartilage. Expression of HAS-ses and HA-binding proteins and receptors such as HAPLN1 and CD44 increases in young and aged PACAP KO mice. Furthermore, the presence of HA is elevated in the articular cartilage during aging. Coincidently, in aged synovial cavity, decreased expression of HA was detected (Temple-Wong et al. 2016). Increased serum HA level was shown in aged population which can be a diagnostic sign of OA (Inoue et al. 2011). Moreover, aggrecanase can induce the HA release from aged articular cartilage which can enhance the destruction of the cartilage matrix during degenerative joint diseases such as OA (Chockalingam et al. 2004). Increased MMP activity was detected in the ECM of cardiac muscle tissue during aging (Meschiari et al. 2017), but we showed a PACAP-dependent reduction of MMPs in mechanical overload of chondrogenic cell cultures (Szentleleky et al. 2019). RHAMM is an intracellular HA-binding receptor which can be activated by ERK (Katona et al. 2016). Its expression is reduced in aged PACAP KO animals, suggesting that intracellular HA function is decreased as it was demonstrated in skin aging (Tzellos et al. 2009). Therefore, the appearance of HA around the chondrons can reflect on imbalanced HA distribution and may be a sign of ECM degradation in PACAP KO knee joints. Previously, we demonstrated that PACAP addition resulted in a reduced expression of hyaluronidases and aggrecenases in stress reactions (Szentleleky et al. 2019). We suppose that chondrocytes cannot precisely control aggrecanase activation in PACAP KO mice. In aged cartilage, the elevated aggrecanase activity is proven to trigger HA release into the synovial cavity. Our results indicate that PACAP is a key molecule in maintaining cartilage matrix and keeping the balance between matrix production and degradation (Fig. 7) (Juhasz et al. 2015b; Szentleleky et al. 2019). Furthermore, the absence of PACAP probably triggers an early aging process which shows the characteristic signs of OA (Fig. 7).

In addition, we also detected increased expression and presence of collagen type I and X in aged articular cartilages. The expression of these collagens is stronger in PACAP gene–deficient mice. In intervertebral disc degradation an increased collagen type X was shown (Hristova et al. 2011) or age related elevation of collagen type X was demonstrated in bone and ligament healing (Wang et al. 2006). Furthermore, aging induced the expression elevation of collagen type I and X in mandibular condylar cartilage (Inoue et al. 2002). We also published that addition of PACAP can induce the normalization of collagen type X expression in mechanical overload (Juhasz et al. 2015b). Additionally, PACAP deficiency increased collagen type I expression in femurs (Jozsa et al. 2018). According to our previous results, PACAP can act on cartilage ECM structure via activation hedgehog signaling (Juhasz et al. 2015b). The absence of PACAP disturbs signaling crosstalks of hedgehog activation, subsequently augments the expression of bone-specific collagen expressions and enhances cartilage matrix calcification. These findings further confirm that the lack of PACAP triggers an accelerated cartilage aging and the appearance of non-specific collagens in cartilage may cause OA-like cartilage matrix formation (Fig. 7).

In conclusion, we provided evidence that PACAP has an important function in proper cartilage matrix production in young animals as it seems to keep the balance between matrix production and matrix degradation (Fig. 7). The lack of the neuropeptide disturbs normal ECM composition of articular cartilage leading to early signs of aging in this tissue, which ultimately can cause a degenerative joint disease (Fig. 7).

## Abbreviations

cAMP: cyclic adenosine monophosphate
CD44: cluster of differentiation 44
CNS: central nervous system
CREB: cAMP response element-binding protein
Chst11: carbohydrate sulphotransferase 11
dNTP: deoxynucleotide triphosphate
ECM: extracellular matrix
EDTA: ethylene diamine tetra-acetic acid
EGTA: ethylene glycol-bis(β-aminoethyl ether)-*N*,*N*,*N*′,*N*′-tetraacetic acid
ERK: extracellular signal–regulated kinase
FBS: foetal bovine serum
FGF: fibroblast growth factor
GAG: glycosaminoglycan
HA: hyaluronic acid
HAPLN: hyaluronan and proteoglycan link protein
HAS: hyaluronan synthase
HH: hedgehog
MAPK: mitogen-activated protein kinase
OA: osteoarthritis
PAC1: pituitary adenylate cyclase–activating polypeptide type I receptor
PACAP: pituitary adenylate cyclase–activating polypeptide
PBS: phosphate-buffered saline
PBST: phosphate-buffered saline supplemented with 1% Tween-20
PG: proteoglycan
PLC: phospholipase C
PKA: protein kinase A
PKC: protein kinase C
PP2A and B: protein phosphatase 2A and B
RT-PCR: reverse transcription followed by polymerase chain reaction
RHAMM: receptor for hyaluronan mediated motility
Sox: SRY-related HMG-BOX gene
TGFβ: transforming growth factor-β
VIP: vasoactive intestinal peptide
VPAC: vasoactive intestinal peptide receptor
WNT: wingless related integration site
